# The mechanochemical Scholl reaction as a versatile synthesis tool for the solvent-free generation of microporous polymers†

**DOI:** 10.1039/d0ra05279e

**Published:** 2020-07-06

**Authors:** Annika Krusenbaum, Sven Grätz, Sarah Bimmermann, Stefanie Hutsch, Lars Borchardt

**Affiliations:** Anorganische Chemie I, Ruhr-Universität Bochum Universitätsstraße 150 44801 Bochum Germany lars.borchardt@ruhr-uni-bochum.de

## Abstract

Herein we report the mechanochemical Scholl polymerization of 1,3,5-triphenylbenzene in a high speed ball mill. The reaction is conducted solvent-free, solely using solid FeCl_3_. The resulting porous polymer was obtained in >99% yield after very short reaction times of only 5 minutes and exhibits a high specific surface area of 658 m^2^ g^−1^, which could be further enhanced up to 990 m^2^ g^−1^ by liquid assisted grinding. Within this study we illuminate the origin of porosity by investigating the impact of various milling parameters and milling materials, temperature and pressure, and different liquids for LAG as well as post polymer milling. Finally we expand the procedure to different monomers and mills, to present the mechanochemical Scholl reaction as a versatile synthesis tool for porous polymers.

## Abstract 2

Herein we report the mechanochemical Scholl polymerization of 1,3,5-triphenylbenzene in a high speed ball mill.

## Graphical abstract

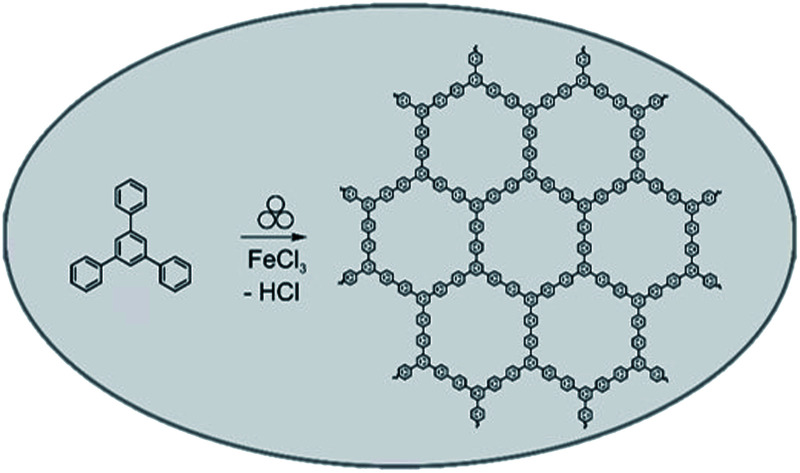

## Introduction

Microporous materials are ubiquitously applied in various fields of industrial relevance such as in heterogeneous catalysis,^1,2^ gas-^3^ and ionic charge storage,^4,5^ or membrane separation.^6^ As these materials are defined as solids containing interconnected pores with small diameters of less than 2 nm, they usually possess high specific surface areas of up to 3000 m^2^ g^−1^.^7^ While inorganic microporous materials such as porous metal oxides^8,9^ or zeolites^10^ have already been explored thoroughly within the last decades, purely organic frameworks gain increasing research interest nowadays. Within this material group, particularly porous organic polymers (POPs) are of high significance, as they feature certain characteristics like high surface areas with adjustable pore sizes and enhanced physiochemical stability.^11,12^ They can be subdivided into crystalline covalent organic frameworks (COFs)^12,13^ and amorphous porous aromatic frameworks (PAFs),^14–16^ hyper-crosslinked polymers (HCPs),^17,18^ polymers of intrinsic microporosity (PIMs)^1,19^ and conjugated microporous polymers (CMPs).^6,20^

The synthesis of POPs is mainly achieved by solvent-based approaches, like for example by Friedel–Crafts alkylations,^21^ metal-catalysed reactions^22,23^*e.g.* Suzuki or Sonogashira–Hagihara coupling reactions, Schiff base reactions^24,25^ and cyclotrimerization reactions.^26,27^ In addition to these reactions, one important tool for the coupling of aryl systems is the Lewis acid-mediated Scholl reaction.^28–31^ Although this reaction is known for certain advantages, such as the lacking requirement for expensive catalysts, starting materials or external cross linkers, there are still certain drawbacks to bypass.^11^ The wet chemical approach mainly suffers from the low solubility of especially larger precursor molecules, which has to be circumvented by the introduction of solubilizing groups and results in additional synthesis steps. Furthermore, instant precipitation of the products can lead to a low degree of polymerization.

Mechanochemical synthesis concepts, such as high energy ball milling, display a versatile tool to circumvent the aforementioned drawbacks and enhance the syntheses sustainability.^32–34^ The transfer of mechanical energy from the colliding balls to the involved particles in a high speed ball mill results in a chemical reaction.^35–37^ Therefore, no solvents are utilized, which makes it possible to bypass the solubility issues of larger monomers and minimize the generated waste.^38–40^ Recently, we have proven feasibility and sustainability of this approach applying it to the synthesis of nanographenes, and highly porous thiophene polymers.^41,42^ Furthermore, by now several mechanochemical processes for the solvent free generation of porous polymers are known. Examples are the mechanochemical synthesis of polymers of intrinsic microporosity (PIMs),^43^ which are especially useful as membranes in gas separation, of COFs,^44^ known for their diverse applicability in photoelectric devices, in catalysis or for gas storage, and of covalent triazine frameworks (CTFs),^45^ capable for selective CO_2_ capture, or to serve as electrode material in lithium–sulfur batteries or super capacitors.^46^

Within this study, we showcase how microporous polymers can be synthesized *via* a mechanochemical Scholl reaction in the solvent-free environment of a ball mill. During the reaction, the starting material 1,3,5-triphenylbenzene was intensely milled with the solid Lewis acid FeCl_3_. The systematic variation of the milling parameters promoted the rapid synthesis of the desired porous polymer within very short reaction times of 5 minutes and in yields of up to >99%. Additionally, it was possible to further enhance the generated surface area up to 1000 m^2^ g^−1^ by liquid-assisted grinding (LAG). To determine the impact of the added liquid and the HCl evolution, temperature and pressure measurements were performed *in situ* during the milling process. Overall, the mechanochemical synthesis approach provides enormous advantages in comparison to the wet chemical approach, as there is no need for harsh reaction conditions or hazardous solvents, although the Lewis acid could be substituted with an even more sustainable alternative in future. Nevertheless, the short reaction time and the easy scalability renders the mechanochemical Scholl coupling reaction as promising synthesis pathway for the generation of porous organic polymers.

## Experimental section

In a typical synthesis approach, 0.540 g (1.77 mmol, 1 eq.) 1,3,5-triphenylbenzene and 3.460 g (21.33 mmol, 12 eq.) FeCl_3_ were brought to reaction in a 50 ml ZrO_2_ grinding jar, implemented in a Retsch MM500 mixer mill. As standard reaction parameters, a milling time of 5 min with a frequency of 30 Hz and 22*Ø* = 10 mm ZrO_2_ milling balls (each ball with an average weight of 3.2 g) was chosen. Subsequent to the completed reaction, the resulting solid was washed out of the jar with water. This mixture was filtered and washed with acetone to yield a black powder, which was dried at 80 °C overnight.

In another approach, the polymer synthesis was transferred to a Fritsch Pulverisette 7 premium line planetary ball mill to measure the pressure and temperature during the reaction inside the reaction vessel with a GTM (gas pressure and temperature measurement) system. For the synthesis, 22*Ø* = 10 mm ZrO_2_ milling balls (average weight of 3.2 g per ball) were implemented in a 45 ml ZrO_2_ milling beaker. To achieve a comparability between the products obtained by the two different milling types, the reaction was performed at 800 rpm within 5 minutes, while the amount of implemented chemicals was kept constant. In addition to this, the workup was conducted in the same fashion as aforementioned.

The porosity of the synthesized porous polymer was investigated by nitrogen physisorption measurements, performed on a Quantachrome Quadrasorb instrument at 77 K. Prior to the measurement, all samples were activated at 353 K for 24 h under vacuum. For physisorption measurements, high purity gases were used (N_2_: 99.999%). The specific surface areas (SSA_BET_) were determined using the BET (Brunauer, Emmett, Teller) equation with the help of a micropore BET assistant. Additionally, total pore volumes were estimated using the adsorption branch at *p*/*p*_0_ = 0.95 and pore size distributions were calculated by the DFT (Density Functional Theory) method for slit, cylindrical and sphere pores. In addition to this, the polymer was characterized by solid state ^13^C CP-MAS NMR experiments, which were performed on the Bruker DSX 400 spectrometer, equipped with a VTN double resonance max. 35 kHz 2.5 mm MAS ^1^H XBB probe head. The samples were rotated at 10 kHz and measured with ramped ^1^H–^13^C cross polarization. As reference for the peak assignments, an adamantane probe was utilized. Infrared spectroscopy (IR) was carried out on a SHIMADZU IRSpirit Fourier transform infrared spectrometer equipped with a single reflection ATR unit. Thermogravimetric analysis (TGA) and simultaneous differential thermal analysis (DTA) were performed on a Seiko TG 6200/SII. For each measurement, 10 mg of the respective compound were filled into an aluminium crucible. Afterwards, the crucible was immersed in a constant nitrogen flow (300 ml min^−1^; 99.999%) inside the machine and measured with a heating rate of 5 °C min^−1^ in a temperature regime between 30 °C and 550 °C. Elemental Analysis (EA) was carried out by the Elementar vario MIRCRO-cube instrument from Elementar. The composition of the sample was determined with respect to the carbon, nitrogen, hydrogen and sulphur content. Rutherford backscattering spectrometry (RBS) was performed at RUBION (Central Unit for Ion Beams and Radionuclides at Ruhr-University Bochum). Therefore, a 2.0 MeV ^4^He^+^ ion beam (intensity 20–40 nA) was directed towards the sample at 7° and the backscattered particles were detected at 160° (Si detector, resolution = 16 keV). Powder X-ray diffraction (PXRD) was investigated with a Bruker D2 PHASER spectrometer with CuKα (1.54184 Å) radiation. UV/VIS absorption spectra were recorded with the Jasco V-670 spectrometer between 300–2500 nm. SEM images were recorded, using a high-resolution scanning electron microscope (JEOL 7500F) at 5 kV and with a Hitachi SU8020 SEM equipped with a secondary electron (SE) detector at 2 kV.

## Results and discussion

### Mechanochemical Scholl reaction of 1,3,5-triphenylbenzene

The mechanochemical Scholl polymerisation of 1,3,5-triphenylbenzene was carried out exemplarily under the described conditions and serves as reference system. The resulting product is a porous polymer and will be declared as PP1 (porous polymer 1) from now on. In order to ensure the reproducibility of this reaction, we have repeated the standard reaction five times. During this investigation, the specific surface area (SSA_BET_) was varying between 640–658 m^2^ g^−1^, which corresponds to a discrepancy of 2.7%, while the yield was alternating between 99 and >99%.

**Fig. 1:**
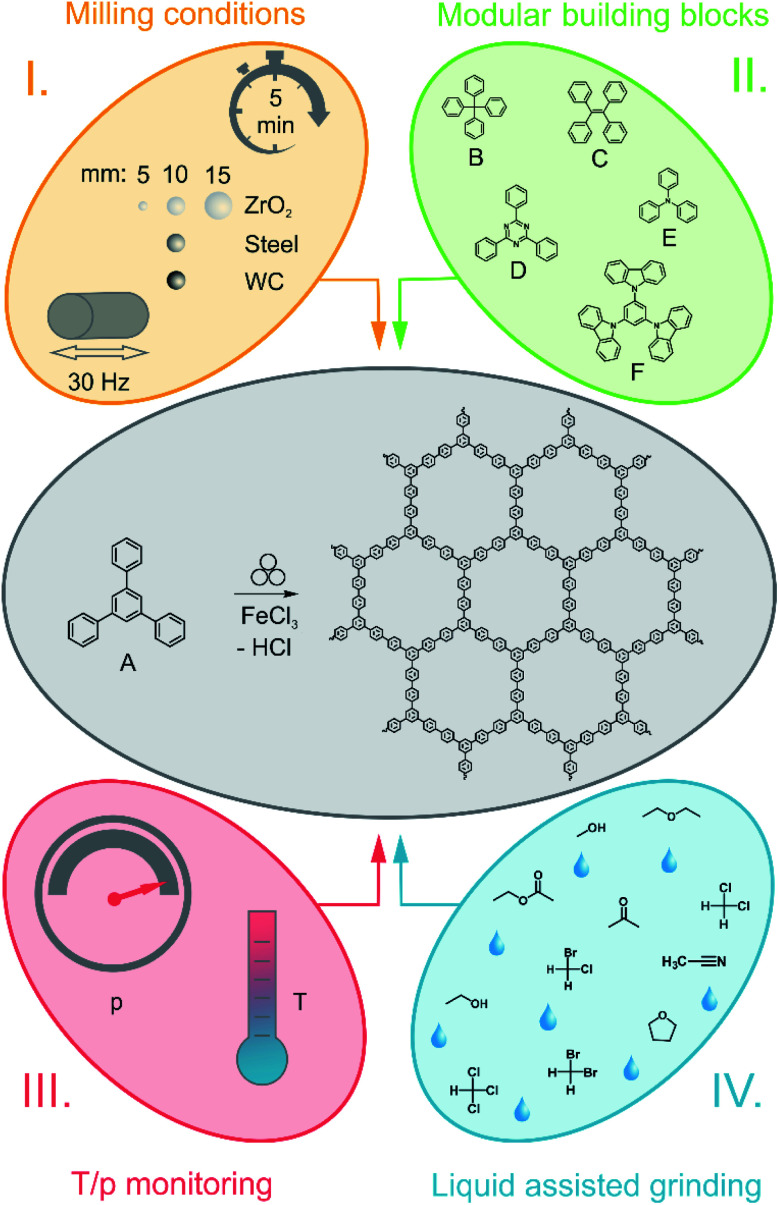
Schematic overview over the parameter variation for the Scholl reaction. The middle part shows the standard reaction in a MM500 mixer mill at 30 Hz for 5 min. For the reaction, the monomer A (1,3,5-triphenylbenzene) is used. I. Systematic variation of milling parameters, as for instance the time, frequency and size and density of the milling material. II. Variation of monomers used for the Scholl reaction. For the reaction, the monomers B (tetraphenylmethane), C (tetraphenylethylene), D (2,4,6-triphenylbenzene-1,3,5-triazine), E (triphenylamine) and F (1,3,5-tris(*N*-carbazolyl)benzene) were used. III. The variation of the temperature during the reaction. Temperature and pressure were recorded during the reaction using a gas pressure and temperature measurement (GTM) system. IV. The Scholl reaction with liquid assisted grinding (LAG).

**Fig. 2:**
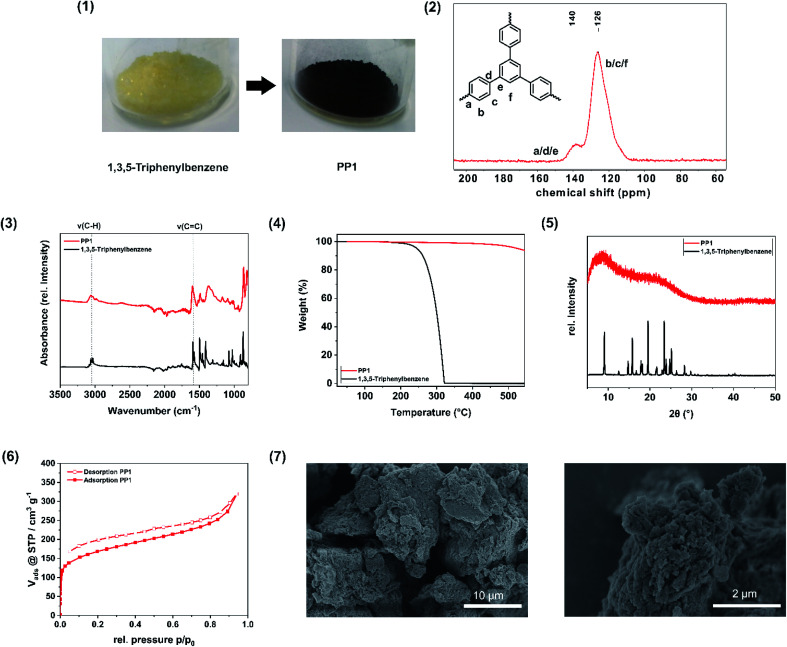
Analysis of the porous polymer PP1. (1) Image of the monomer in comparison to the polymer. (2) ^13^C cross-polarization magic-angle spinning (CP-MAS) NMR of the porous polymer PP1. The peaks are assigned to the spectrum. (3) FT-IR spectra of PP1 (red) and 1,3,5-triphenylbenzene (black). The absorption band of the C–H and C=C stretching vibration is highlighted. (4) Thermogravimetric analysis (TGA) of PP1 (red) in comparison to the monomer 1,3,5-triphenylbenzene (black). (5) Powder X-ray diffractogram (PXRD) of PP1 (red) compared with the monomer 1,3,5-triphenylbenzene (black), showing the amorphous behaviour of the polymer. (6) Nitrogen physisorption isotherm of the porous polymer PP1 exhibiting a polymer-like swelling behaviour. (7) SEM image of the sample PP1 with a magnitude of 5000 (left) and 30 000 (right).

During the polymerization, a colour change from pale yellow (monomer) to dark brown (polymer) occurred (Fig. 2(1)). The CP-MAS NMR spectrum of the polymer (Fig. 2(2)) exhibits two broad resonance peaks at *δ* = 140 ppm and *δ* = 126 ppm, which can be assigned to the coupled aromatic carbons a, d and e and to the non-coupled aromatic carbons b, c and f, respectively, and were already observed for the 1,3,5-triphenylbenzene polymer synthesized from hot CHCl_3_ under inert gas atmosphere and AlCl_3_ as Lewis acid.^30^ FT-IR investigations (Fig. 2(3)) show the intact C–H(benzene) and C=C(benzene) vibrations in the monomer 1,3,5-triphenylbenzene and in the polymer PP1, as in the region of 3056 cm^−1^ a weak absorption band is visible, which is attributed to the C–H(benzene) stretching vibration, while at 1592 cm^−1^ the C=C(benzene) stretching vibration can be seen, which is assignable to the aromatic ring skeleton vibration. The observations are consistent with the FT-IR spectrum of 1,3,5-triphenylbenzene polymer synthesized by the solution based approach.^30^ The Scholl polymer shows high thermal stability of up to 500 °C (Fig. 2(4)). In comparison to the crystalline monomer 1,3,5-triphenylbenzene, the polymer PP1 exhibits an amorphous structure, which can be attributed to multiple stacking faults of the polymeric networks in the bulk material (Fig. 2(5)), to a distortion of the structure through the biphenyl axis, or to interpenetration. Moreover, possible cross-linking and side reactions of the forming polymer skeleton, particularly at longer reaction times, lead to structural irregularities, hence to a deviation from the schematical structure (Fig. 1) and thus explains its amorphous structure. To verify the successful intermolecular coupling reactions, UV/VIS spectroscopy was performed to neglect the occurrence of competing intramolecular reactions, which would result in the formation of fluorene units. These would feature two broad absorption bands in the UV/VIS at 303 nm and at 314 nm, which are not present in the spectrum of PP1 (Fig. S3†).^47^ The nitrogen physisorption (Fig. 2(6)) isotherms can be assigned to IUPAC type I, which is characteristic for microporous materials. With use of the Brunauer–Emmett–Teller-equation it was possible to determine the specific surface area of the PP1 to 658 m^2^ g^−1^. Additionally, the total pore volume was calculated from the adsorption branch at *p*/*p*_0_ = 0.95 to 0.53 cm^3^ g^−1^ and the pore width was found to be 0.64 nm with a fitting error of 0.3%. In comparison to the solution based approach (exhibiting a surface area of 1254 m^2^ g^−1^), the specific surface area is smaller, nevertheless it was possible to further enhance the SSA_BET_ by liquid assisted grinding, yielding a comparable value (see: Impact of liquid-assisted grinding). SEM images of the porous polymer reveal the agglomeration of smaller, unevenly shaped particles to bigger flakes (Fig. 2(7)). To determine the elemental composition of the synthesised polymer, elemental analysis (EA) was performed. The obtained values are in a range with the expected, which indicates a high purity of the polymer (Table S3†). To investigate the degree of contamination, Rutherford backscattering spectrometry was performed. On the basis of 61 at% C and 38 at% H, the total contamination of Cl (0.94 at%) and of Fe (0.04 at%) was found to be below 1 at%. Due to this, the workup with water and acetone was fond to be promising to remove the traces of unreacted monomer as well as of the Lewis acid FeCl_3_.

### Influence of the milling parameters frequency, time and milling material

During the process development, the systematic variation of milling parameters of the reference system PP1 was a key technique to obtain the highest possible yield with the highest possible SSA_BET_ within the shortest possible time. Therefore, the milling time was varied in a first attempt, while the frequency was kept constant at 30 Hz. The results are presented in Table 1 as PP1–PP7.

**Table 1:** Yields, specific surface areas (SSA_BET_) and pore volume for the polymers obtained at different milling times and frequencies. The total pore volume *V*_total_ was calculated from the N_2_ isotherm at *p*/*p*_0_ = 0.95, 77 K

Polymer	Material	Time (min)	Frequency (Hz)	Yield (%)	SSA_BET_ (m^2^ g^−1^)	*V*_total_ (cm^3^ g^−1^)
PP1	ZrO_2_ (10 mm)	5	30	>99	658	0.53
PP2	ZrO_2_ (10 mm)	1	30	19	87	0.17
PP3	ZrO_2_ (10 mm)	2	30	51	61	0.09
PP4	ZrO_2_ (10 mm)	10	30	>99	348	0.28
PP5	ZrO_2_ (10 mm)	15	30	>99	505	0.40
PP6	ZrO_2_ (10 mm)	30	30	>99	568	0.61
PP7	ZrO_2_ (10 mm)	60	30	>99	421	0.33
PP8	ZrO_2_ (10 mm)	5	10	26	17	0.06
PP9	ZrO_2_ (10 mm)	5	20	44	87	0.13
PP10	ZrO_2_ (10 mm)	5	25	75	111	0.18
PP11	ZrO_2_ (10 mm)	5	35	>99	273	0.24
PP12	WC (10 mm)	5	30	>99	581	0.43
PP13	Steel (10 mm)	5	30	>99	499	0.36
PP14	ZrO_2_ (5 mm)	5	30	83	285	0.12
PP15	ZrO_2_ (15 mm)	5	30	>99	457	0.36

The Scholl polymerization of 1,3,5-triphenylbenzene is incomplete after very short reaction times of 1 or 2 minutes (PP2 and PP3). Within this time, the formation of the polymer was only achieved with 19% and 51% yield and low specific surface areas of 87 m^2^ g^−1^ and 61 m^2^ g^−1^, respectively. During the variation of milling time, the reaction was most promising for short reaction times of 5 minutes, as the polymer PP1 was already obtainable in >99% yield. Furthermore, this short reaction exhibits the highest SSA_BET_ of 658 m^2^ g^−1^. Longer milling lead to a partial degradation of the porosity, probably due to the high energy input.

The mechanochemical Scholl polymerization is very sensitive towards the energy input, as the high energy transferred from the milling balls to the particles inside the grinding jar at 35 Hz (*i.e.* only an increase of 5 Hz compared to PP1) results in a severe degradation of the porosity (sample PP11 in Table 1). However, also lower frequencies are not favourable, as the lower energy impact leads to an incomplete reaction with yields far below 100% (PP8–PP10). Due to this, the optimal frequency for the mechanochemical Scholl reaction in a MM500 mixer mill was determined to be 30 Hz (PP1).

The density of the milling material is an important factor for the generation of porous polymers. If the density is too low (*e.g.* Si_3_N_4_), the energy impact might not be sufficient, which hinders a fast reaction necessary for the formation of the pores. Very high density (*e.g.* WC) milling material is not only providing a sufficient amount of energy to the system, but also leads to a heating of the milling jar, which increases the pressure inside the vessel. Nevertheless, the energy impact might also be too high and destroy the readily formed polymers. To proof this suggestion, 22*Ø* = 10 mm WC or steel balls were implemented in a steel beaker under standard conditions (sample PP12 and PP13 in Table 1). Similar to the mechanochemical Scholl reaction at a higher frequency or for longer milling times, the use of higher density materials leads to a degradation of the surface area. Therefore, ZrO_2_ milling balls with a medium density were found to be perfectly suitable for our approach.

In a last approach, the ball size of the ZrO_2_ balls was varied to *Ø* = 5 mm and to *Ø* = 15 mm (sample PP14 and PP15 in Table 1). To ensure comparability, the same total ball masses were used. This corresponds to 174 ZrO_2_ milling balls of *Ø* = 5 mm and 6 ZrO_2_ milling balls of *Ø* = 15 mm, respectively. Again, *Ø* = 10 mm sized balls appear to be optimal, with respect to the porosity of the obtained polymer.

To investigate how the high energy input affects the porosity of the product, we conducted post polymer milling for 30 minutes at 30 Hz. Therefore, the porous polymer PP1 was milled with 12 eq. of FeCl_3_ in one attempt and with the same amount of an inert bulk material (NaCl) in another approach. During the milling process with FeCl_3_, the surface area was degraded to 560 m^2^ g^−1^, which corresponds to 85% of the former polymer. For the milling with NaCl as bulk material, the surface area changed to 432 m^2^ g^−1^, which equals 66% of the starting material. This confirms that the milling itself is degrading the porosity of the formed polymer and reinforces the need of short but intense milling protocols for the synthesis of porous polymers.

### Monomers impact

To explore the origin of porosity, the impact of the monomer was examined. For the investigation, six different monomers were utilized in a standard reaction procedure, while the reaction parameters were kept constant (addition of 12 eq. FeCl_3_, 30 Hz, 5 min). The different monomers are summarized in Table 2 as A–F.

**Table 2:** Yields, specific surface areas (SSA_BET_) and total pore volumes (*V*_total_) for the Scholl polymerization of different monomers

Monomer	Polymer	Yield (%)	SSA_BET_ (m^2^ g^−1^)	*V*_total_ (cm^3^ g^−1^)
A	PP1	>99	658	0.53
B	PP16	12	225	0.24
C	PP17	86	88	0.24
D	PP18	78	n.p.	—
E	PP19	48	161	0.56
F	PP20	>99	1408	0.95

The monomer exchange led to a broad range of porosity of the resulting polymers from non-porous (PP18) to highly porous (PP20) materials. We believe the electron density of the respective aryl compounds to impact the Scholl reaction activity and thus the development of porosity. Electron deficient systems such as 2,4,6-triphenyl-1,3,5-triazine (monomer D), polymerize in yields of 78% and remain non-porous while electron rich aryl systems such as 1,3,5-triphenylbenzene (monomer A) or 1,3,5-tris(*N*-carbazolyl)benzene (monomer F) convert to porous structures with >99% yield. The high SSA_BET_ of the reference system PP1 of 658 m^2^ g^−1^ was solely exceeded by polymer PP20, which was synthesized from 1,3,5-tris(*N*-carbazolyl)benzene (monomer F) and exhibits a SSA_BET_ of 1408 m^2^ g^−1^ (Table 2).

**Fig. 3:**
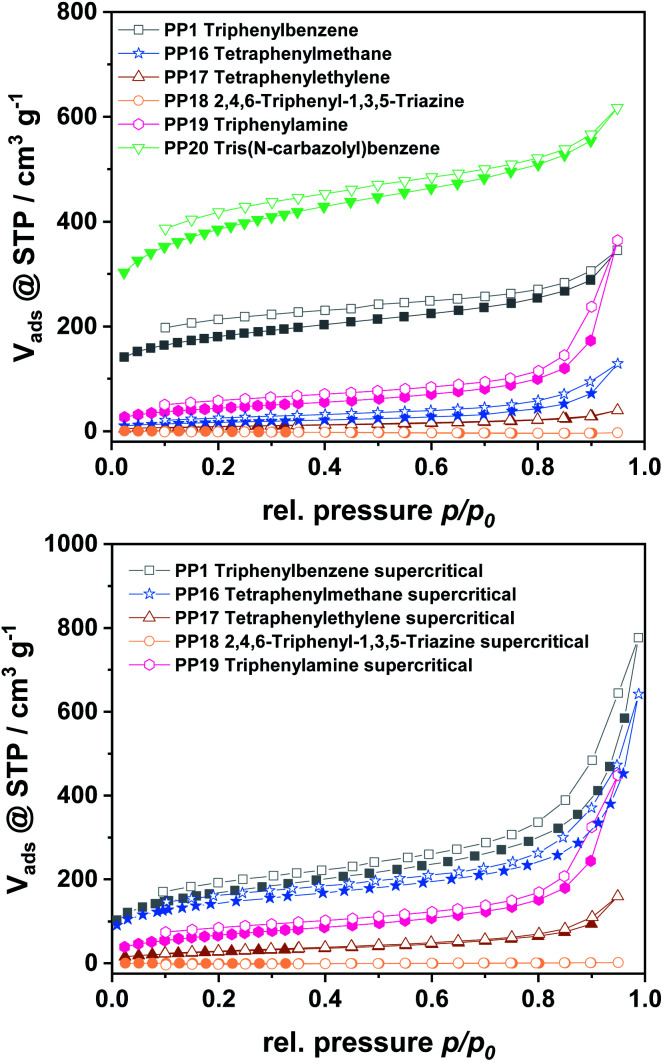
N_2_ physisorption isotherms for polymers dried at 80 °C (top) and for supercritically dried polymers (bottom).

For all polymers, characteristic type I isotherms were observed during nitrogen physisorption at 77 K (Fig. 3), which are representative for microporous materials. The isotherms of the polymers gained from monomer C and E furthermore feature a high nitrogen uptake at *p*/*p*_0_ = 0.95, which is attributed to a high inter-particular space within the sample. All isotherms feature a certain non-reversibility of the adsorption and desorption branch, which is due to the swelling-behaviour of the amorphous polymers. For a deeper insight into the pore formation, the polymers obtained by the different monomers were also dried supercritically to prevent pore collapse during the drying procedure. Due to this it was possible to expand the obtained surface areas by a factor of 1.5–2, as the original pore structure is kept intact during drying.

### The impact of temperature and pressure on the reaction

The collision of milling balls with the grinding vessel walls and each other results in a step by step heating of the milling vessel.

To investigate the impact of the temperature on the mechanochemical Scholl reaction, the reference system was milled at different temperatures. Since a direct regulation of the milling temperature is hardly feasible at the moment we filled and closed the steel milling vessels at room temperature and then transferred them into an oven or refrigerator overnight to achieve a homogenous temperature inside the vessels. Since the reaction time is short, we assumed that the temperature we achieved with external heating or cooling is not changing too much during the milling process, while the milling itself leads to a stepwise increase of the vessel temperature. However, as this temperature increase due to the milling itself is occurring for every reaction, it can be neglected in the given case.

It seems that the reaction is proceeding slower at lower temperatures, leading to lower yields and smaller SSA_BET_ (174 m^2^ g^−1^, PP21). At elevated temperatures higher surface areas could be achieved (657 m^2^ g^−1^, PP26) up to an external temperature of 100 °C. The temperature probably also has an indirect impact on the system, as a higher temperature leads to a further pressure increase inside the milling jar (Table 3).

**Table 3:** Yield, specific surface areas (SSA_BET_) and total pore volume (*V*_total_) for the milling of the reference system at different temperatures inside steel vessels

Polymer	Temperature (°C)	Yield (%)	SSA_BET_ (m^2^ g^−1^)	*V*_total_ (g cm^−3^)
PP21	−50	68	174	0.36
PP22	−20	89	195	0.19
PP23	0	95	359	0.38
PP13	RT	>99	499	0.36
PP24	50	>99	595	0.53
PP25	75	>99	568	0.47
PP26	100	>99	657	0.44
PP27	125	>99	522	0.41

**Fig. 4:**
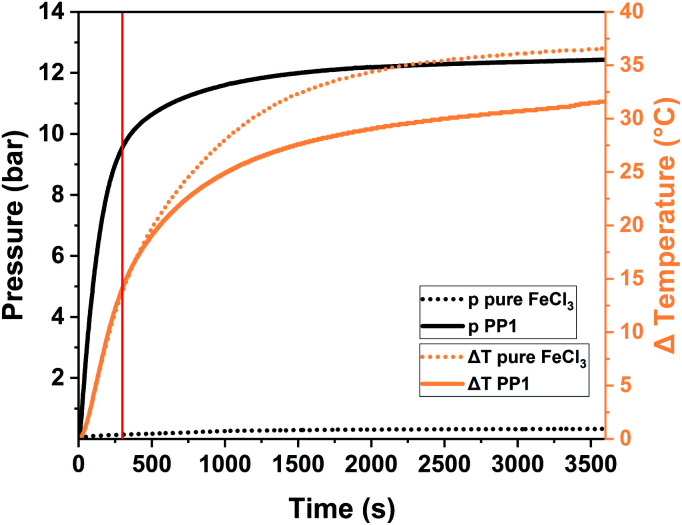
Pressure and temperature profile of the Scholl reaction of the sample PP1 (solid line) in comparison to the grinding of pure FeCl_3_ (dotted line). The standard reaction time of 5 minutes is assigned to the spectrum as red, solid line.

To exhibit the impact of pressure on the system, we had to transfer the reaction to a Fritsch Pulverisette 7 premium line planetary ball mill (P7) operated with a gas pressure and temperature measurement (GTM) system at 800 rpm. Transferring the reaction from a vibrational to a planetary ball mill was possible as the polymers obtained from both mills are comparable among each other (for further information please see Table S2†). For this analysis the milling time was elongated to up to one hour in order to be able to assess the whole pressure and temperature development. For PP1 the temperature is increasing to 37 °C (Δ*T* = 14 °C) after 5 minutes (marked with a red line in Fig. 4) and to 55 °C (Δ*T* = 32 °C) after one hour of milling. While the temperature increase for the milling of pure FeCl_3_ and of the sample PP1 (1 eq. 1,3,5-triphenylbenzene + 12 eq. FeCl_3_) was almost identical, there are huge differences between the pressure developments of the same systems. During the milling of pure FeCl_3_ no reaction takes place, therefore no HCl evolution is occurring. The small increase in pressure to 380 mbar during the milling is attributed to the temperature increase and therefore to the expansion of air captured inside the sealed milling vessel. For the milling of the sample PP1 a steep pressure increase to ∼9.5 bar was observed during the first 5 minutes of the measurements. Afterwards the curve flattens, as the reaction and therefore the evolution of HCl is almost completed. After one hour the overall pressure inside the vessel is settled at ∼12 bar.

### Impact of liquid-assisted grinding

Based on our previous results on hyper-crosslinked polymers, we knew that small quantities of liquid (liquid assisted grinding, LAG) can greatly impact the porosity of polymers.^17^ Therefore, we started to add a varying amount of dichloromethane (DCM) to the grinding vessel.

The addition of small quantities of DCM enhanced the specific surface area of the polymer to more than 1050 m^2^ g^−1^. Larger quantities, *i.e.* 2 ml, of liquid led to a degradation of the porosity again. This behaviour reveals that the enhanced surface area for the addition of small quantities of DCM is not a solvation effect, however the liquid rather serves as catalyst or mediator.

The systematic variation of different liquids added to the grinding jar was conducted to examine the origin of porosity during the reaction (Table 4). In the following, the liquid assisted grinding was performed for up to 1 hour to ensure an adequate *in situ* temperature and pressure monitoring.

**Table 4:** Yields, specific surface areas (SSA_BET_) and total pore volumes (*V*_total_) for liquid assisted grinding with varying amounts of different liquids at 800 rpm in the planetary ball mill P7. The top part (PP28–PP31) exhibits the results for the reaction for 5 minutes and the lower part (PP32–PP42) shows the milling operated for up to 1 hour for *in situ* measurements of pressure and temperature

Polymer	Liquid	Amount (ml)	Yield (%)	SSA_BET_ (m^2^ g^−1^)	*V*_total_ (cm^3^ g^−1^)
PP28	DCM	0.5	>99	1069	0.72
PP29	DCM	1	97	1090	0.73
PP30	DCM	1.5	>99	914	0.64
PP31	DCM	2	>99	733	0.52
PP32	DCM	1	>99	998	0.68
PP33	CHCl_3_	1	>99	845	0.58
PP34	CH_2_Br_2_	1	87	953	0.63
PP35	CH_2_BrCl	1	>99	832	0.57
PP36	Et_2_O	1	90	55	0.01
PP37	EtOH	1	>99	173	0.10
PP38	EtOAc	1	>99	318	0.27
PP39	MeCN	1	>99	72	0.15
PP40	MeOH	1	76	22	0.07
PP41	Acetone	1	>99	39	0.05
PP42	THF	1	>99	71	0.09

At first, we assumed that the liquid may act as solvent for the evolving HCl during the reaction, which would therefore be captured and thus impact the development of porosity of the obtained polymer. However, the solubility of HCl in CHCl_3_ and in DCM is significantly lower than in Et_2_O, which is contrary to the obtained surface areas (PP32, PP33, PP36). In the following, we hypothesized that the boiling point of the liquid impacts pore formation as the vapour may serve as porogen. Again, we can rule out this, as the similarity of the boiling points of DCM (40 °C) and of Et_2_O (35 °C) leads to materials with greatly different surface areas of 998 and 55 m^2^ g^−1^, respectively. Since the porosity of the polymer is greatly enhanced by LAG with CHCl_3_ and DCM, it has to be taken into consideration that these chemicals might not only act as a solvent for HCl, but are part of the reaction process. One suggestion would be a competing Friedel–Crafts reaction, which provides methylene cross-links and enhances the porosity of the polymer. Nevertheless, this was disproved as well, as ^13^C CP-MAS NMR analysis showed no additional signals originating from a methylene cross-linker (see Fig. S5†). During the investigation, solely halogenated liquids resulted in an enlargement of the surface area (PP32–PP35), while the addition of non-halogenated liquids led to a surface area degradation (PP36–PP42). It is presumable that the halogenated liquid and FeCl_3_ associate,^48^ which results in a highly Lewis acidic FeCl_2_^+^ intermediate that is very reactive.^49,50^ This might accelerate the reaction further leading to a higher cross-linking, disorder and thus higher porosity. Interestingly, during the LAG with different liquids, the addition of halogenated liquids gives higher pressures than the addition of non-halogenated liquids, which correlates well with the obtained surface areas (see Fig. S11–S23†). Therefore, we postulate that the high pressure can be attributed to an increased HCl evolution evoked from the highly Lewis acidic FeCl_2_^+^ intermediate, which is increasing the specific surface area of the polymer.

## Conclusion

Herein we reported the mechanochemical Scholl polymerization of 1,3,5-triphenylbenzene with solid FeCl_3_ in a MM500 mixer mill. Our approach is not relying on the use of any solvents, which makes the synthesis unhazardous and more sustainable than the classical Scholl polymerization, performed under the use of inert gas atmosphere and hot CHCl_3_. It was even possible to obtain the desired porous polymer within very short reaction times of only 5 minutes and in yields of up to >99%, which makes the former used multistep synthesis with reaction times of 48 h and a 24 h Soxhlet extraction neglectable. In addition to this, it was possible to transfer the synthesis approach to different monomers, yielding polymers with specific BET surface areas of more than 1400 m^2^ g^−1^. During the study, the pore formation was examined with regards to the drying procedure, the milling-parameters and -materials and the temperature and pressure inside the reaction vessel. Furthermore, the impact of liquid assisted grinding (LAG) was examined with respect to the evolution of HCl during the reaction. Summarizing, the mechanochemical Scholl reaction is a versatile tool for the fast, easy and sustainable synthesis of porous polymers, which are very important in several industrial applications nowadays.

## Conflicts of interest

There are no conflicts to declare.

## Supplementary Material

RA-010-D0RA05279E-s001
