# Supplementary material for: The mechanochemical Scholl reaction as a versatile synthesis tool for the solvent-free generation of microporous polymers
Source: RSC Adv. 2020 Jul 6;10(43):25509–16. doi: 10.1039/d0ra05279e (PMC9055252; doi:10.1039/d0ra05279e)
Supplement: RA-010-D0RA05279E-s001 [file RA-010-D0RA05279E-s001.pdf]

Electronic Supplementary Material (ESI) for RSC Advances.
This journal is © The Royal Society of Chemistry 2020

Electronic Supplementary Material (ESI) for…. This journal is ©

Supporting Information

The mechanochemical Scholl reaction as a versatile synthesis tool for the solvent-free generation of microporous polymers

Annika Krusenbaum,[a] Sven Grätz,[a] Sarah Bimmermann,[a] Stefanie Hutsch,[a] Lars Borchardt[a*]

[a] Anorganische Chemie I, Ruhr-Universität Bochum,
Universitätsstr. 150, 44801 Bochum

Table of Contents

1. Materials 2
2. Milling parameters 2
3. Characterisation 5
3.1. PP1 (reference system) 5
3.2. PP29 (reference system + 1 ml DCM) 8
3.3. Gas pressure and temperature measurements for LAG 11

1. Materials

Iron(III) chloride (anhydrous, Alfa Aesar, 98% purity), 1,3,5-Triphenylbenzene (Aldrich, 97 % purity), Tetraphenylmethane (BLDpharm, 97% purity), Tetraphenylethylene (chemPUR, 98% purity), 2,4,6-Triphenyl-1,3,5-Triazine (TCI, >98% purity), Triphenylamine (TCI, >98% purity), 1,3,5-Tris(N-carbazolyl)benzene (Aldrich, >97% purity). All chemicals were purchased as received.

Zirconium oxide milling balls (Type ZY-S) in Ø = 5 mm (average weight of 0.40 g), Ø = 10 mm (average weight of 3.2 g) and Ø = 15 mm (average weight of 11.73 g) were purchased from Sigmund Lindner GmbH. Tungsten carbide milling balls (YG6X, G10 surface) in Ø = 10 mm with an average weight of 7.20 g were purchased from Zhuzhou Good Cemented Carbide Co., Ltd. Tempered steel milling balls (1.4125, AISI 440C) in Ø = 10 mm with an average weight of 4.02 g were purchased from TIS Wälzkörpertechnologie GmbH.

2. Milling parameters

Table S1: Overview over the yield, specific surface area (SSA_{BET}) and the total pore volume (V_{total}) for various polymers obtained by the use of different milling parameters.

Polymer	Monomer	Material	Time (s)	Frequency (Hz/rpm) mill	Liquid Amount (ml)	Temperature (°C)	Yield (%)	SSA_{BET} (m^2/g)	V_{total} (g/cm^3)
**PP1**	A	ZrO_2 (10 mm)	5	30 MM500	/	RT	> 99	658	0.53
**PP2**	A	ZrO_2 (10 mm)	1	30 MM500	/	RT	19	87	0.17
**PP3**	A	ZrO_2 (10 mm)	2	30 MM500	/	RT	51	61	0.09
**PP4**	A	ZrO_2 (10 mm)	10	30 MM500	/	RT	> 99	348	0.28
**PP5**	A	ZrO_2 (10 mm)	15	30 MM500	/	RT	> 99	505	0.40
**PP6**	A	ZrO_2 (10 mm)	30	30 MM500	/	RT	> 99	568	0.61
**PP7**	A	ZrO_2 (10 mm)	60	30 MM500	/	RT	> 99	421	0.33

**PP8**	A	ZrO_2 (10 mm)	5	10<br>MM500	/	RT	26	17	0.06
**PP9**	A	ZrO_2 (10 mm)	5	20<br>MM500	/	RT	44	87	0.13
**PP10**	A	ZrO_2 (10 mm)	5	25<br>MM500	/	RT	75	111	0.18
**PP11**	A	ZrO_2 (10 mm)	5	35<br>MM500	/	RT	> 99	273	0.24
**PP12**	A	WC (10 mm)	5	30<br>MM500	/	RT	> 99	581	0.43
**PP13**	A	Steel (10 mm)	5	30<br>MM500	/	RT	> 99	499	0.36
**PP14**	A	ZrO_2 (5 mm)	5	30<br>MM500	/	RT	83	285	0.12
**PP15**	A	ZrO_2 (15 mm)	5	30<br>MM500	/	RT	> 99	457	0.36
**PP16**	B	ZrO_2 (10 mm)	5	30<br>MM500	/	RT	12	225	0.24
**PP17**	C	ZrO_2 (10 mm)	5	30<br>MM500	/	RT	86	88	0.24
**PP18**	D	ZrO_2 (10 mm)	5	30<br>MM500	/	RT	78	n.p.	-
**PP19**	E	ZrO_2 (10 mm)	5	30<br>MM500	/	RT	48	161	0.56
**PP20**	F	ZrO_2 (10 mm)	5	30<br>MM500	/	RT	> 99	1408	0.95
**PP21**	A	Steel (10 mm)	5	30<br>MM500	/	- 50	68	174	0.36
**PP22**	A	Steel (10 mm)	5	30<br>MM500	/	- 20	89	195	0.19
**PP23**	A	Steel (10 mm)	5	30<br>MM500	/	0	95	359	0.38
**PP24**	A	Steel (10 mm)	5	30<br>MM500	/	50	> 99	595	0.53
**PP25**	A	Steel (10 mm)	5	30<br>MM500	/	75	> 99	568	0.47
**PP26**	A	Steel (10 mm)	5	30<br>MM500	/	100	> 99	657	0.44
**PP27**	A	Steel (10 mm)	5	30<br>MM500	/	125	> 99	522	0.41
**PP28**	A	ZrO_2 (10 mm)	5	800<br>P7	DCM<br>0.5 ml	RT	> 99	1069	0.72

**PP29**	A	ZrO_2 (10 mm)	5	800 P7	DCM 1 ml	RT	97	1090	0.73
**PP30**	A	ZrO_2 (10 mm)	5	800 P7	DCM 1.5 ml	RT	> 99	914	0.64
**PP31**	A	ZrO_2 (10 mm)	5	800 P7	DCM 2 ml	RT	> 99	733	0.52
**PP32**	A	ZrO_2 (10 mm)	60	800 P7	DCM 1 ml	RT	> 99	998	0.68
**PP33**	A	ZrO_2 (10 mm)	60	800 P7	$CHCl_3$ 1 ml	RT	> 99	845	0.58
**PP34**	A	ZrO_2 (10 mm)	60	800 P7	CH_2Br_2 1 ml	RT	87	953	0.63
**PP35**	A	ZrO_2 (10 mm)	60	800 P7	CH_2BrCl 1 ml	RT	> 99	832	0.57
**PP36**	A	ZrO_2 (10 mm)	60	800 P7	Et_2O 1 ml	RT	90	55	0.01
**PP37**	A	ZrO_2 (10 mm)	60	800 P7	EtOH 1 ml	RT	> 99	173	0.10
**PP38**	A	ZrO_2 (10 mm)	60	800 P7	EtOAc 1 ml	RT	> 99	318	0.27
**PP39**	A	ZrO_2 (10 mm)	60	800 P7	MeCN 1 ml	RT	> 99	72	0.15
**PP40**	A	ZrO_2 (10 mm)	60	800 P7	MeOH 1 ml	RT	76	22	0.07
**PP41**	A	ZrO_2 (10 mm)	60	800 P7	Acetone 1 ml	RT	> 99	39	0.05
**PP42**	A	ZrO_2 (10 mm)	60	800 P7	THF 1 ml	RT	> 99	71	0.09
**Post polymer $FeCl_3$**	Polymer	ZrO_2 (10 mm)	30	30 MM500	/	RT	> 99	560	0.39
**Post polymer NaCl**	Polymer	ZrO_2 (10 mm)	30	30 MM500	/	RT	> 99	432	0.43

Table S2: Comparison between the yields, specific surface areas (SSA_{BET}) and total pore volumes (V_{total}) obtained for the standard reaction (Reaction of 1,3,5-Triphenylbenzene; 12 eq. $FeCl_3$, 5 min) in the Fritsch Pulverisette 7 premium line planetary ball mill (P7) at 800 rpm and in the Retsch mixer mill MM500 (MM500) at 30 Hz. For the reactions, different amounts of DCM were added.

	**P7**			**MM500**		
	**Yield (%)**	**SSA_{BET} (m^2/g)**	**V_{total} (cm^3/g)**	**Yield (%)**	**SSA_{BET} (m^2/g)**	**V_{total} (cm^3/g)**
**0.5 ml DCM**	> 99	1069	0.72	> 99	976	0.72
**1 ml DCM**	97	1090	0.73	> 99	990	0.69
**1.5 ml DCM**	> 99	914	0.64	> 99	956	0.67
**2 ml DCM**	> 99	733	0.52	> 99	740	0.76*

* sample contains large amounts of interparticular space

3. Characterisation

3.1. PP1 (reference system)

Table S3: Elemental analysis of **PP1**.

	**C (wt.%)**	**H (wt.%)**	**N (wt.%)**	**S (wt.%)**
**Calculated**	95.02	4.98	-	-
**Found**	96.87	2.93	-	-

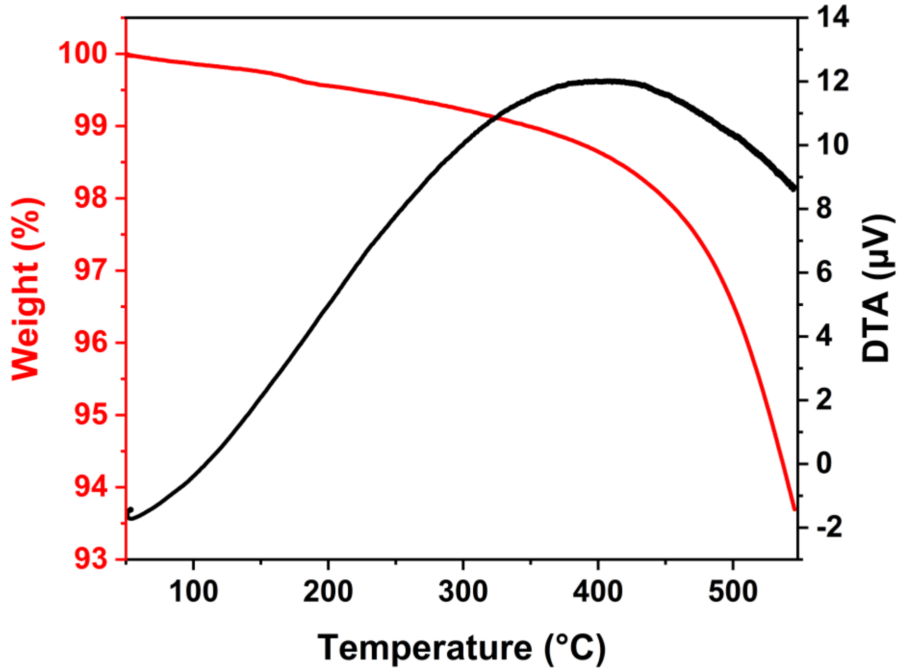

Figure S1: Thermogravimetric analysis (TGA; red) and differential thermal analysis (DTA; black) of the Porous Polymer **PP1**.

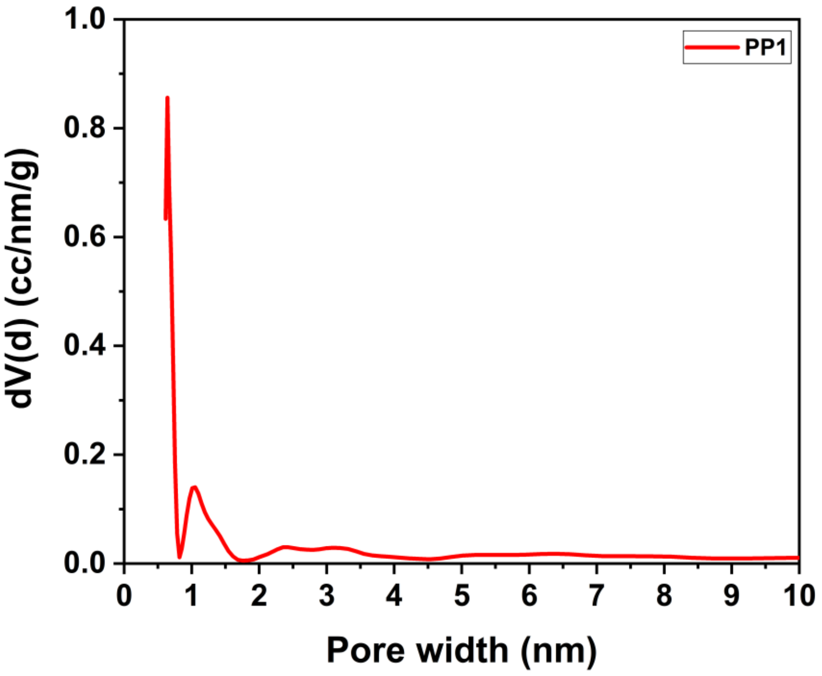

Figure S2: Pore Size distribution (red) of the Porous Polymer **PP1**.

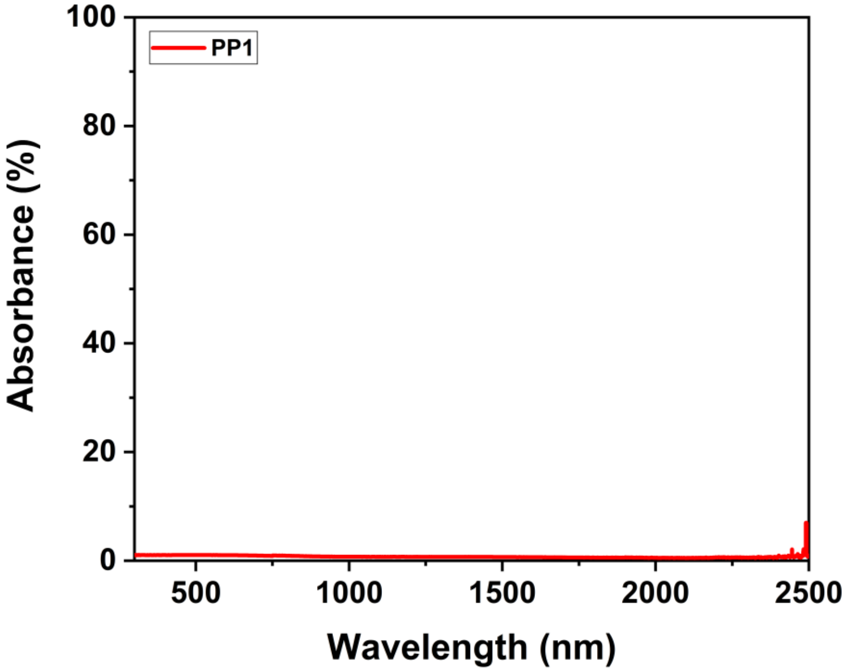

Figure S3: UV/VIS spectrum (red) of the Porous Polymer 1.

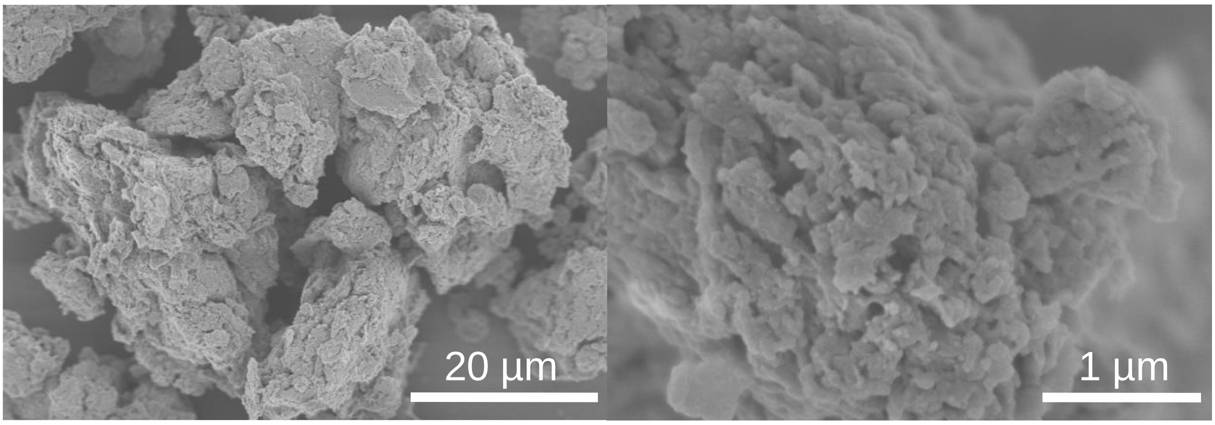

Figure S4: SEM image of the sample **PP1** with a magnitude of 2000 (left) and of 50000 (right).

3.2. PP29 (reference system + 1 ml DCM)

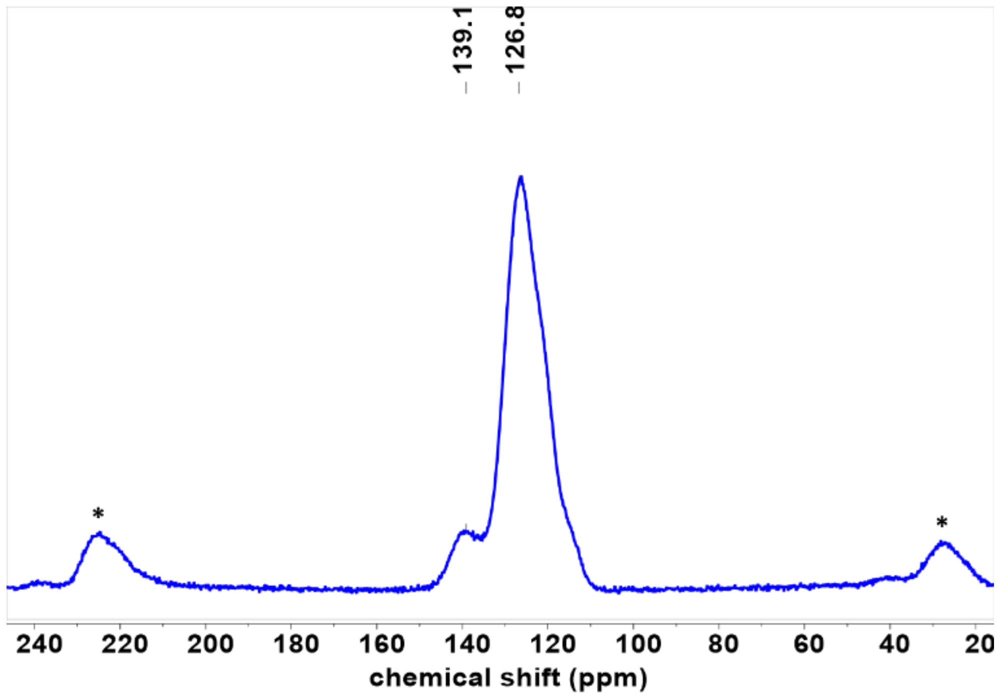

Figure S5: ^{13}C CP-MAS NMR spectrum of **PP29**. The peaks are assigned to the spectrum and the spinning bands are marked with an asterisk.

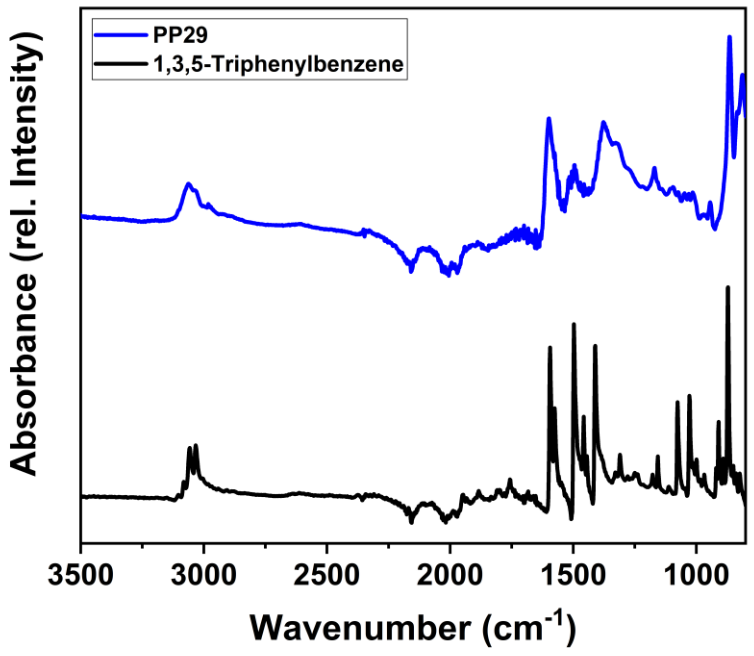

Figure S6: FT-IR spectra of **PP29** (blue) and 1,3,5-Triphenylbenzene (black).

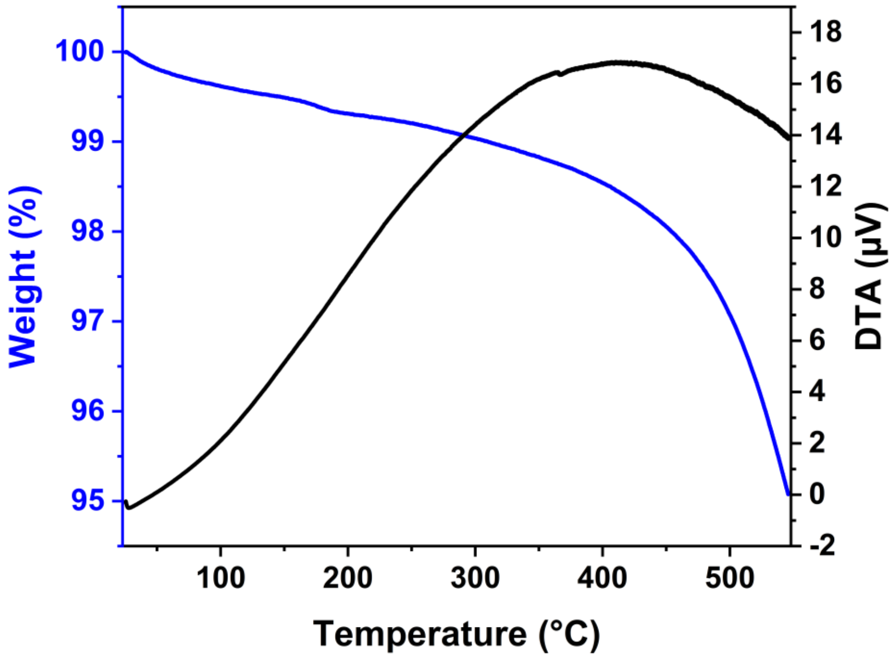

Figure S7: Thermogravimetric analysis (black) and Differential thermal analysis (DTA; red) of the Porous Polymer **PP29**.

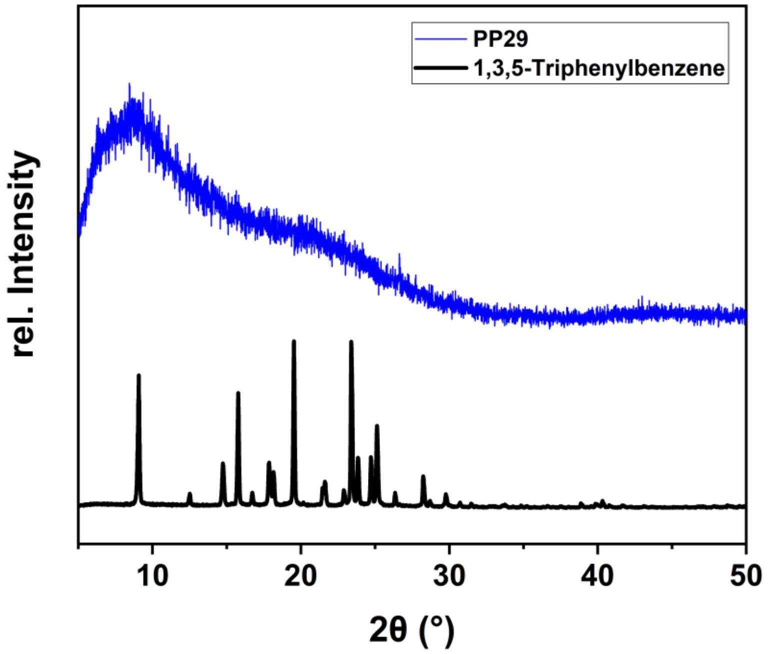

Figure S8: Powder X-ray diffractogram (PXRD) of **PP29** (blue) compared with the monomer 1,3,5-Triphenylbenzene (black), showing the amorphous behaviour of the polymer.

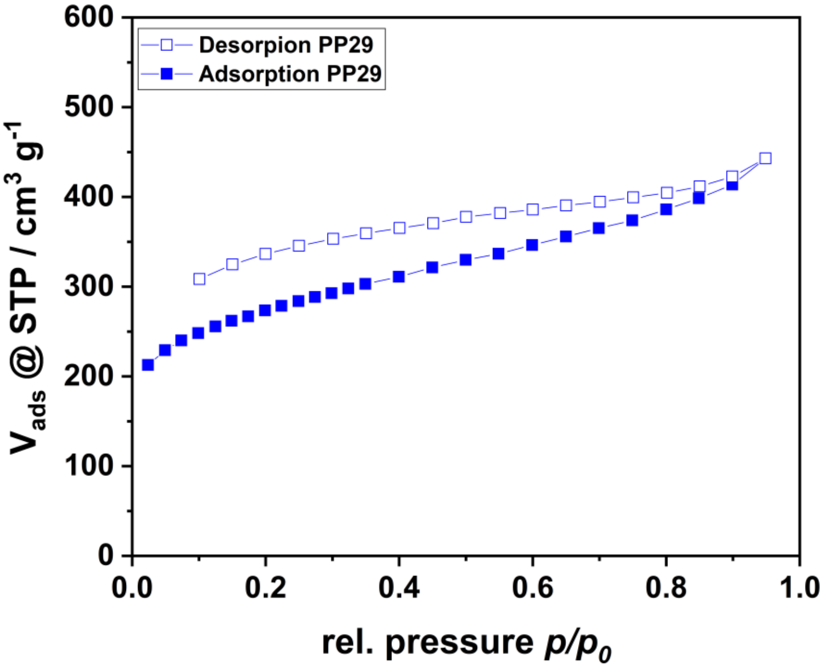

Figure S9: Nitrogen physisorption isotherm IUPAC type I of the porous polymer **PP29** exhibiting a polymer-like swelling behaviour.

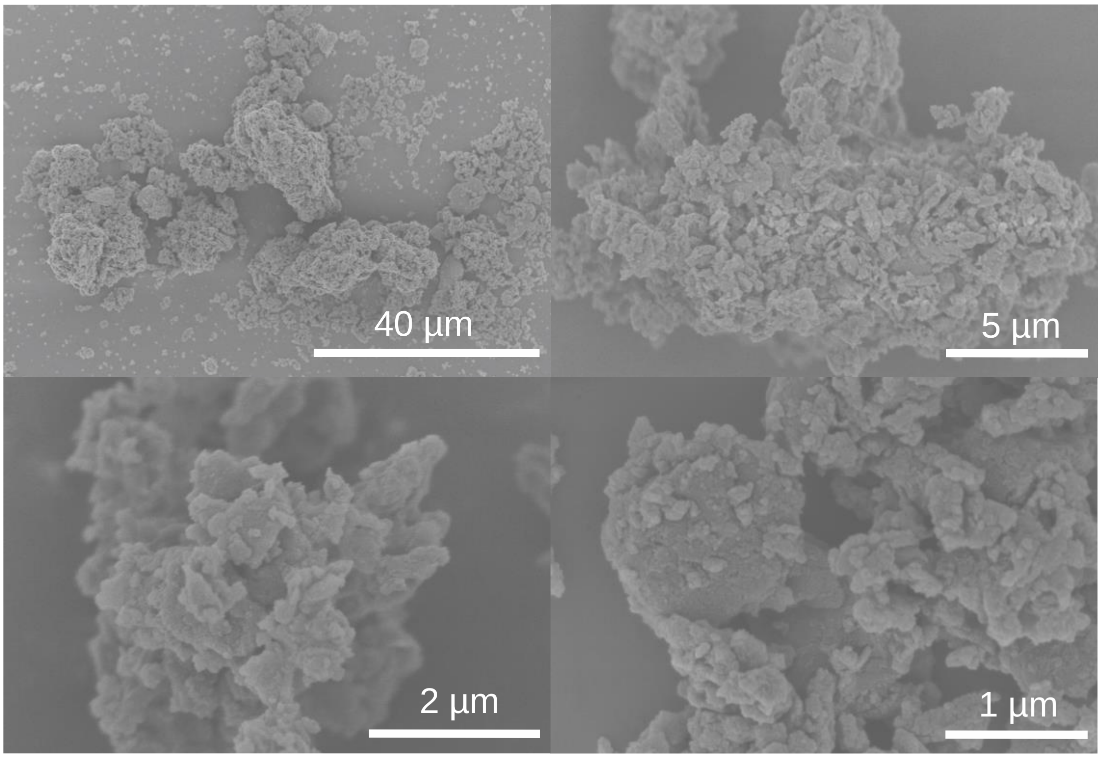

Figure S10: SEM image of the sample **PP29** with a magnitude of 2000 (top, left), of 10000 (top, right), of 30000 (bottom, left) and of 50000 (bottom, right).

3.3. Gas pressure and temperature measurements for LAG

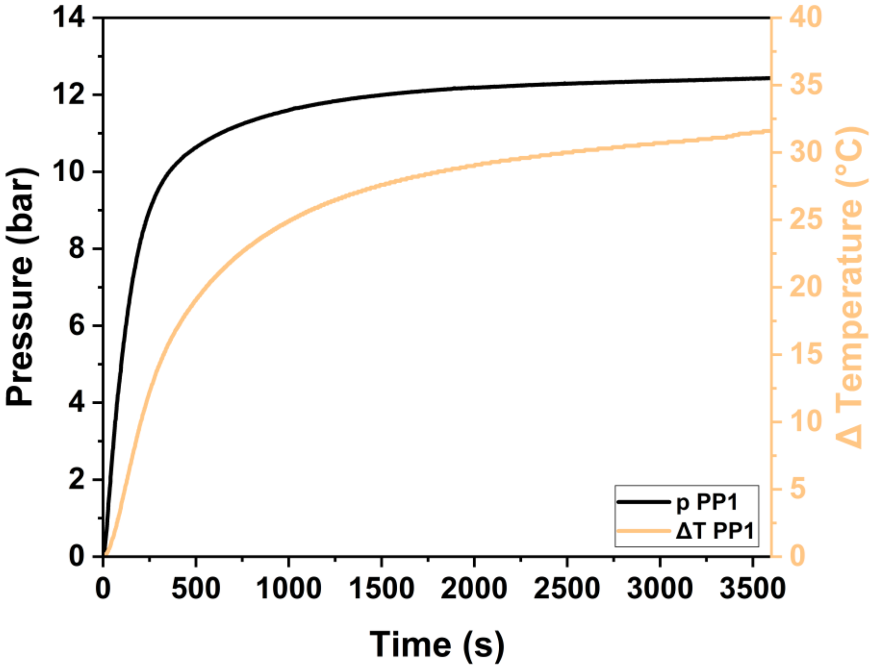

Figure S11: Gas pressure (black) and temperature (beige) measurement for the elongated milling of **PP1**.

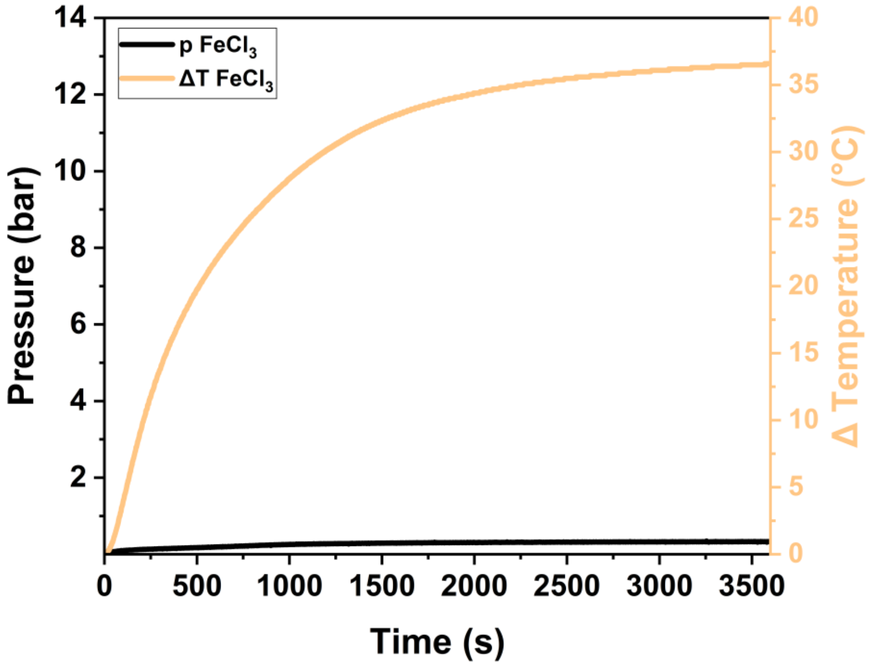

Figure S12: Gas pressure (black) and temperature (beige) measurement for the milling of pure $FeCl_3$.

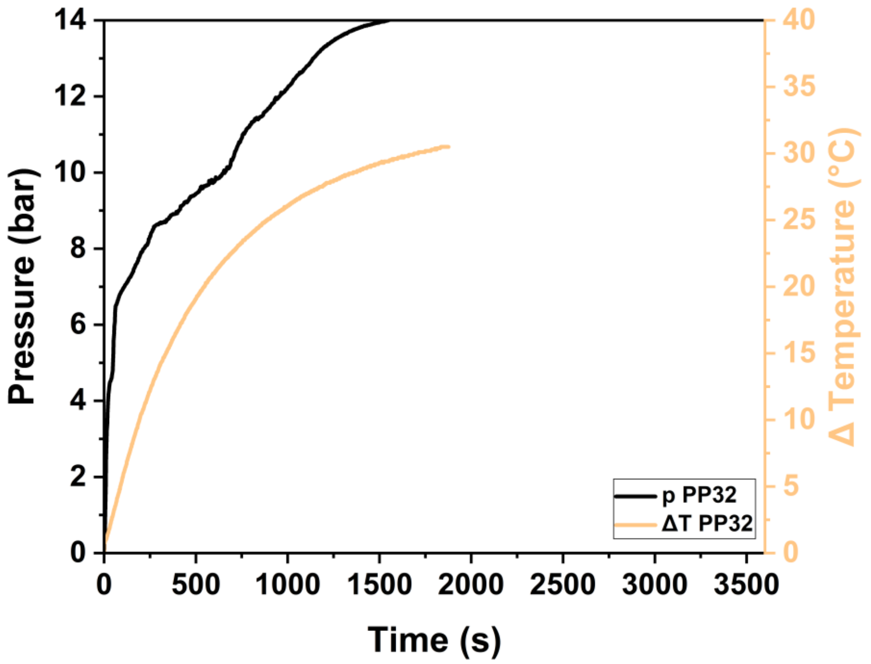

Figure S13: Gas pressure (black) and temperature (beige) measurement for the LAG with 1 ml DCM. A pressure limit of 14 bar was surpassed after ~ 1500 s, which led to milling abortion.

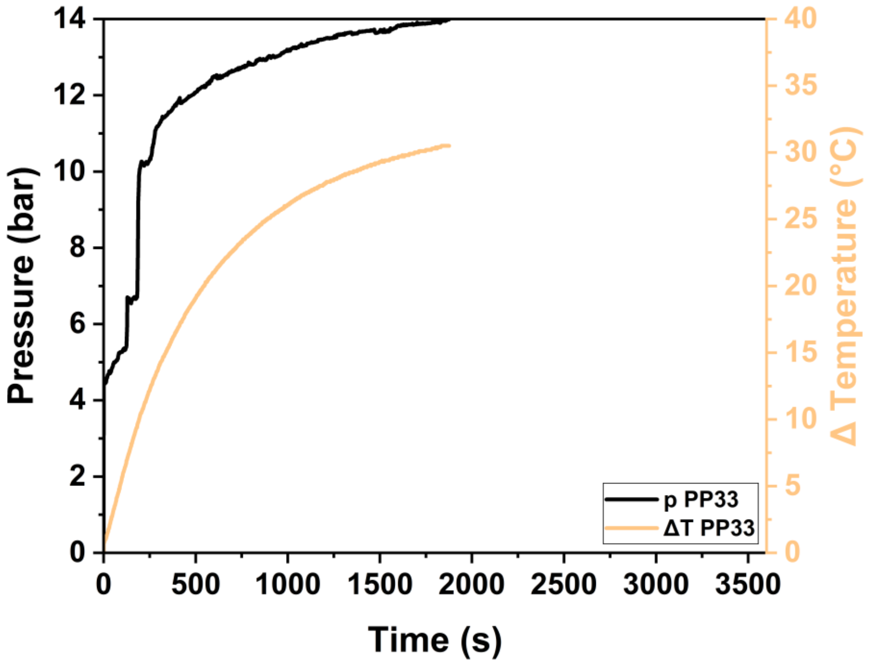

Figure S14: Gas pressure (black) and temperature (beige) measurement for the LAG with 1 ml $CHCl_3$. A pressure limit of 14 bar was surpassed after ~ 2000 s, which led to milling abortion.

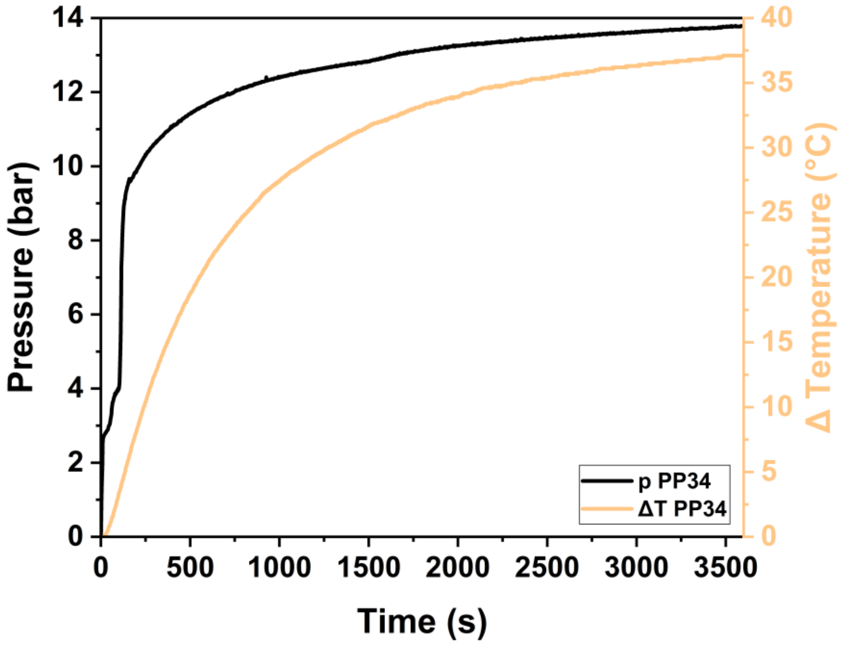

Figure S15: Gas pressure (black) and temperature (beige) measurement for the LAG with 1 ml CH_2Br_2.

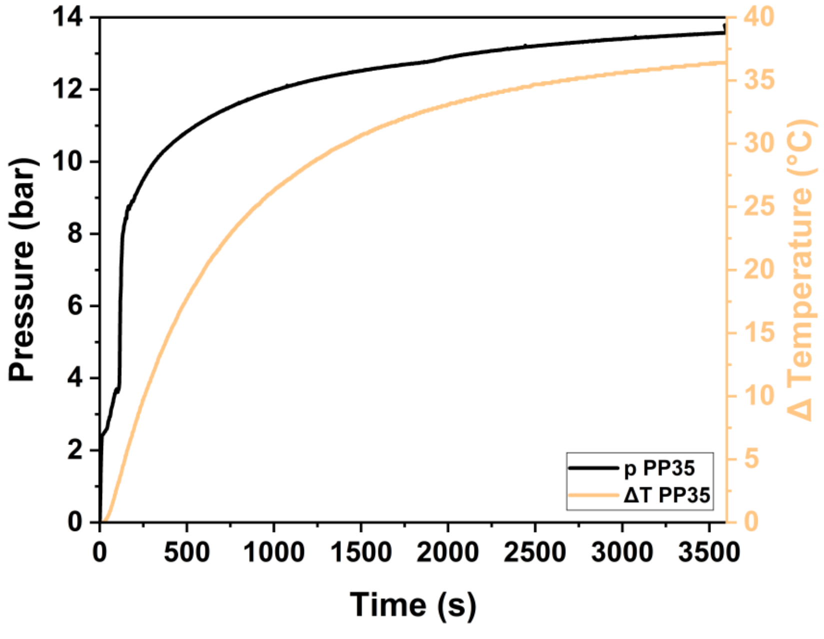

Figure S16: Gas pressure (black) and temperature (beige) measurement for the LAG with 1 ml CH_2BrCl.

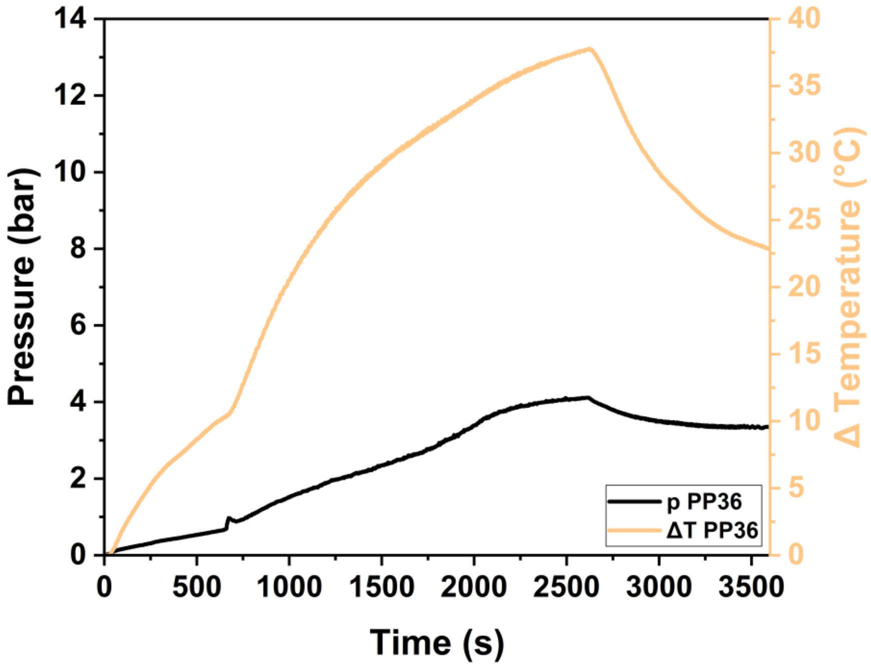

Figure S17: Gas pressure (black) and temperature (beige) measurement for the LAG with 1 ml Et_2O.

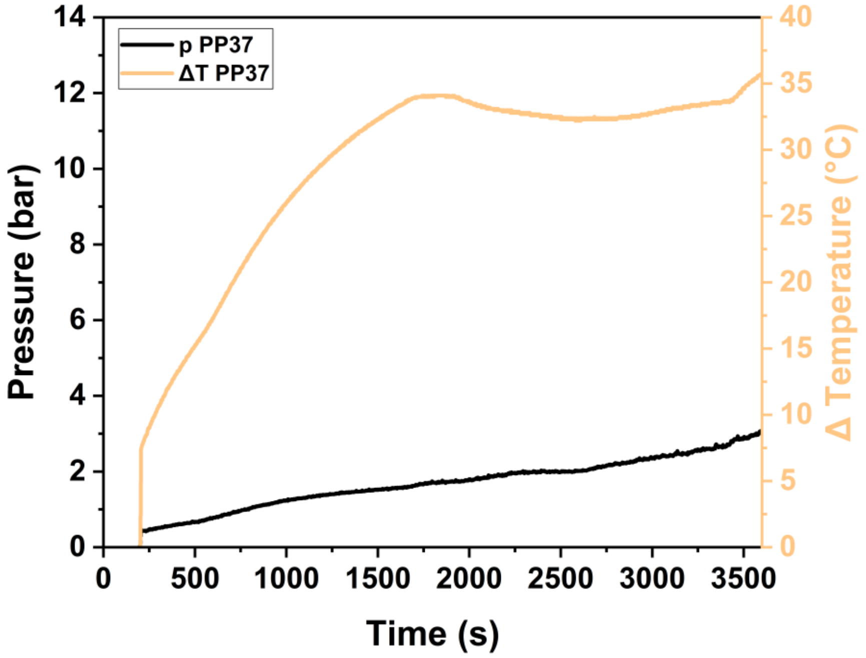

Figure S18: Gas pressure (black) and temperature (beige) measurement for the LAG with 1 ml EtOH.

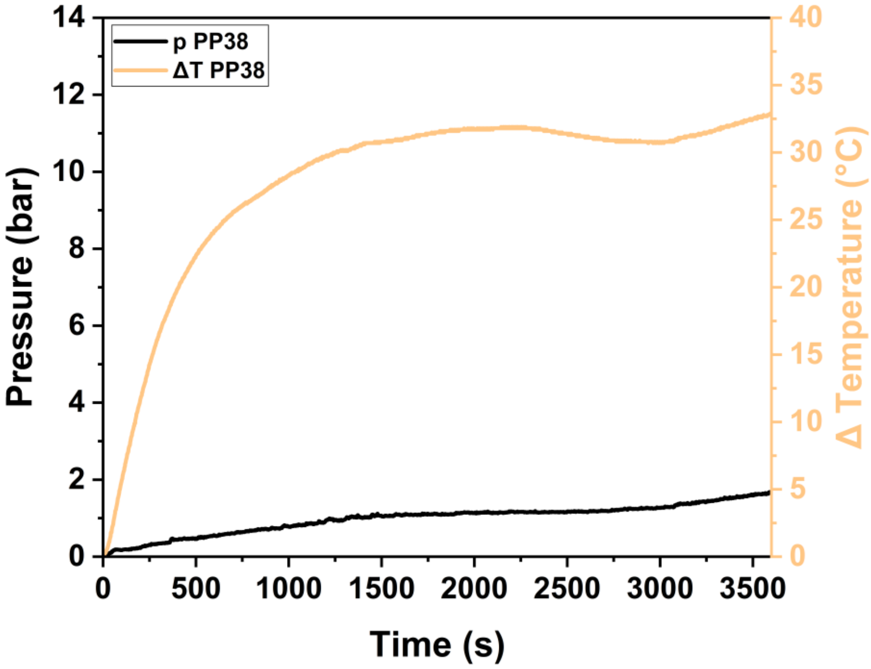

Figure S19: Gas pressure (black) and temperature (beige) measurement for the LAG with 1 ml EtOAc.

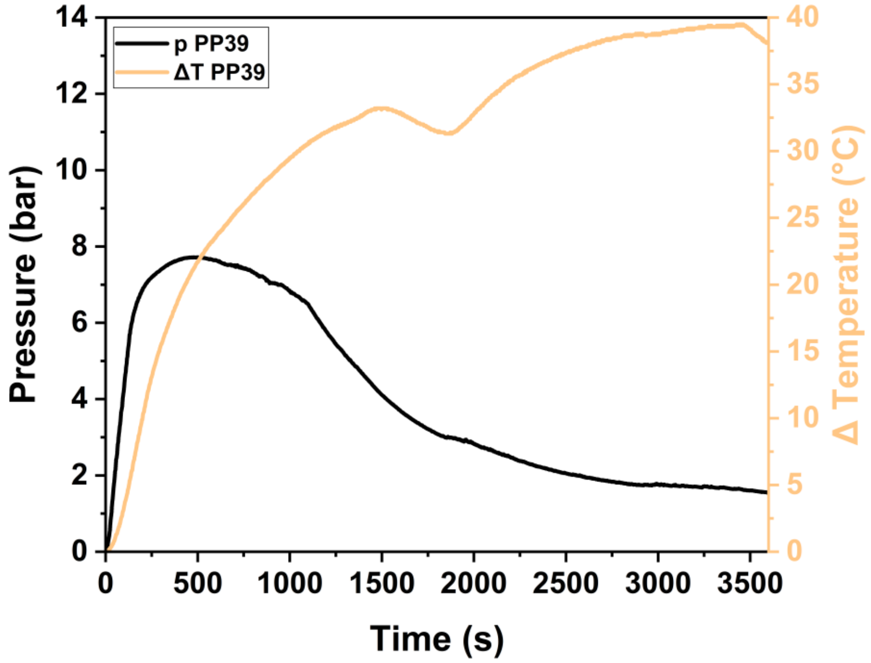

Figure S20: Gas pressure (black) and temperature (beige) measurement for the LAG with 1 ml MeCN.

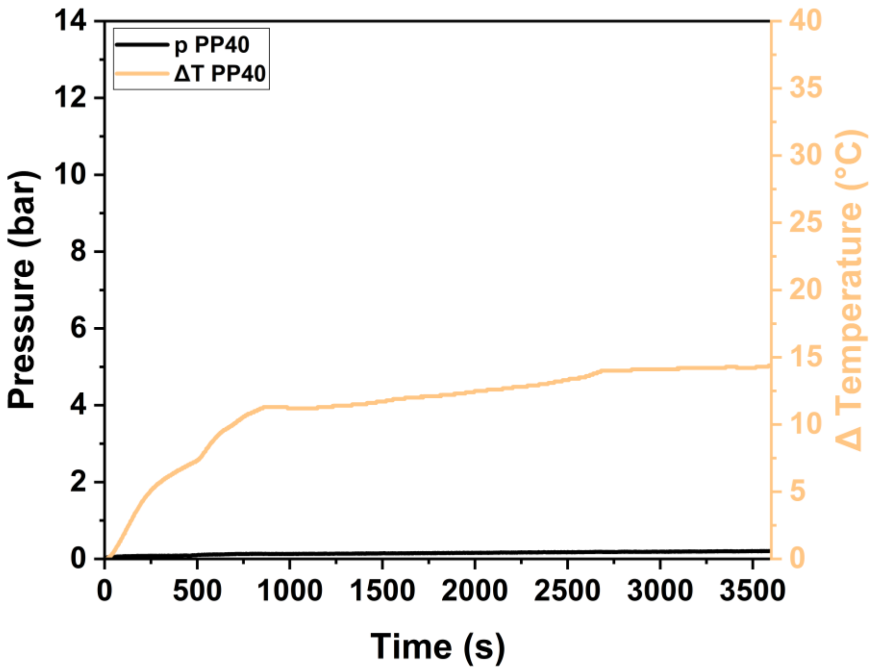

Figure S21: Gas pressure (black) and temperature (beige) measurement for the LAG with 1 ml MeOH. Due to the low temperature increase the reaction was repeated, yielding the same result.

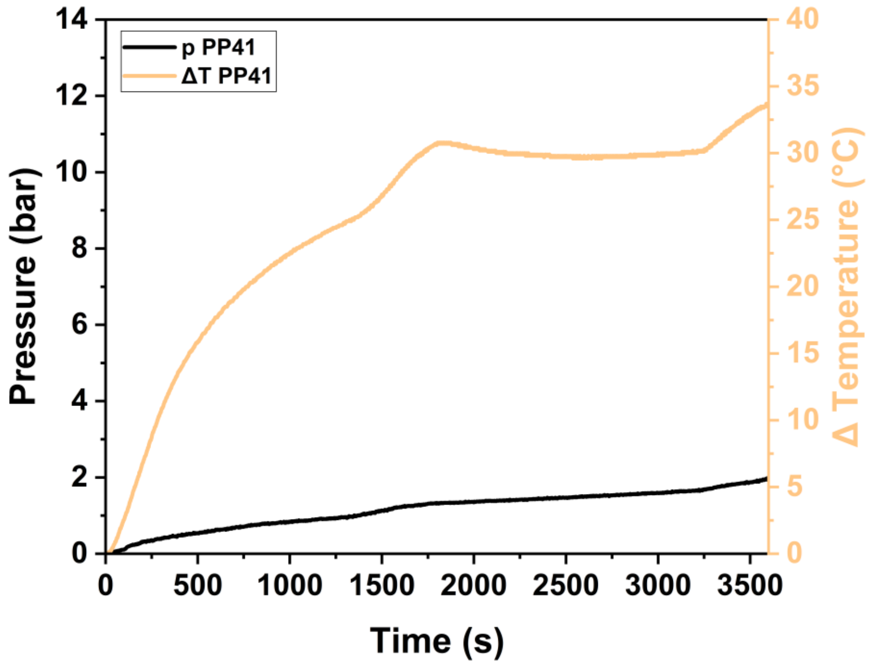

Figure S22: Gas pressure (black) and temperature (beige) measurement for the LAG with 1 ml acetone.

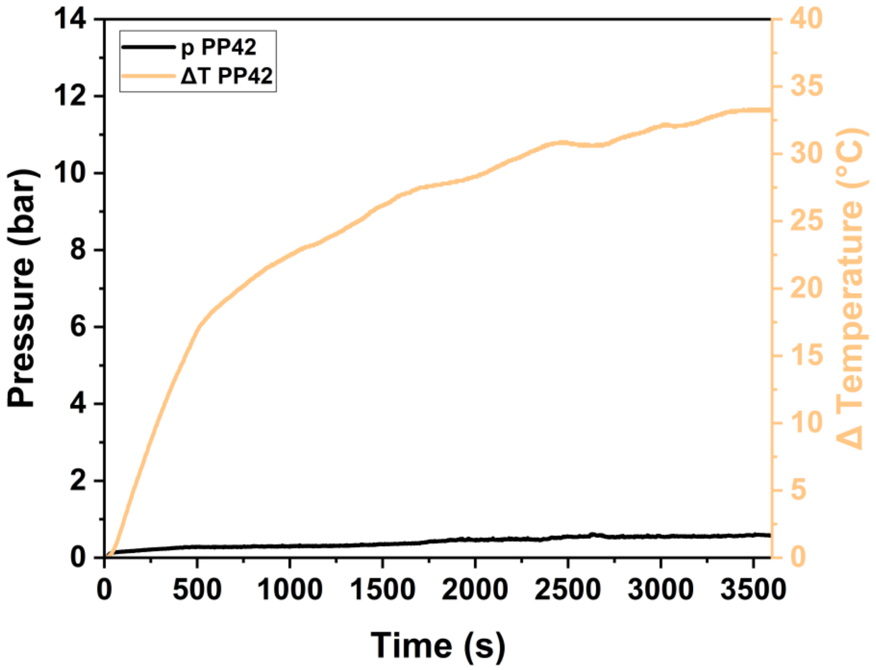

Figure S23: Gas pressure (black) and temperature (beige) measurement for the LAG with 1 ml THF.
